# Integration of Multi-Scale Predictive Tools of Bone Fragility: A Structural and Material Property Perspective

**DOI:** 10.3390/ma18194639

**Published:** 2025-10-09

**Authors:** Muhammad Ateeq, Laura Maria Vergani, Federica Buccino

**Affiliations:** 1 Department of Mechanical Engineering (DMEC), Politecnico di Milano, Via La Masa 1, 20156 Milano, Italy; muhammad.ateeq@polimi.it (M.A.); federica.buccino@polimi.it (F.B.); 2 IRCCS Galeazzi-Sant’Ambrogio, Via Cristina Belgioioso 173, 20157 Milan, Italy

**Keywords:** fragility fractures, predictive tools, multiscale, osteoporosis

## Abstract

Bone fragility represents a significant global health burden, characterized by the deterioration of bone strength, increased brittleness, and heightened fracture susceptibility. Osteoporosis substantially elevates the risk of fragility fractures, the principal clinical manifestation of the disease. Current diagnostic approaches, including biomedical imaging, bone strength assessment, and bone mineral density measurement, are closely linked to identifying bone fragility through various predictive models and tools. Although numerous studies have employed predictors to characterize fragility fractures, few have comprehensively examined the morpho-structural features of bone across multiple hierarchical scales, limiting the ability to fully elucidate the underlying mechanisms of bone fragility. This review summarizes recent advancements in predictive modeling and novel diagnostic tools, focusing on multiscale approaches for assessing bone fragility. We critically evaluate the translational potential of these tools for the early detection of fragility fractures and their clinical application in mitigating fracture risk. Moreover, this study discusses the integration of multiscale predictive methodologies, which promise to enhance early-stage bone fragility detection and potentially prevent severe fractures through timely intervention. Finally, the study reflects on current research limitations, addressing the challenges associated with multiscale predictive modeling of bone fragility, and proposes future directions to refine these tools to improve the accuracy and utility of fragility fracture prediction and prevention strategies.

## 1. Introduction

Bone fragility is a significant global health issue, as the increased risk of fractures can lead to severe consequences, including higher mortality rates, reduced quality of life, loss of independence, and a substantial economic burden. Bone fragility has a significant impact on quality of life by increasing bone loss and reducing bone turnover, which elevates the risk of bone fractures. Traditionally, bone fragility has been linked to a decrease in bone mass, deficiencies in mineralization, or structural abnormalities within the bone matrix [1]. As the population ages, the frequency of fragility fractures is expected to rise, placing a growing socioeconomic burden on healthcare systems worldwide. This growing incidence of fractures, particularly among postmenopausal women, is primarily attributed to age-related changes in bone structure and a decline in bone mass, emphasizing the importance of early detection to prevent fractures and reduce the global burden of bone fragility [2]. Bone fragility is a degenerative condition caused by disrupted homeostasis, leading to decreased bone strength and increased risk of fractures without significant trauma [3].

A primary cause of bone fragility is osteoporosis. The osteoporosis is a progressive, multifactorial systemic skeletal disorder characterized by reduced bone mass, deterioration of bone tissue microarchitecture, and decreased bone strength, leading to an elevated risk of fractures [4]. Osteoporosis contributes to over nine million fractures annually worldwide, with an osteoporotic fracture approximately estimated to occur every three seconds. Approximately one-third of women and one-fifth of men will experience an osteoporotic fracture in their lifetime [5,6]. As life expectancy increases, the prevalence of chronic conditions like osteoporosis is expected to rise correspondingly [7]. The global population affected by osteoporosis and associated fractures has increased significantly since 1990 and continues to rise. For instance, in 2019, approximately 178 million new bone fractures were recorded, which is a 33.4% increase since 1990 [8]. Similarly, it is predicted that between 2030 and 2034, there will be 263.2 million osteoporosis incidence cases worldwide (154.4 million for women and 108.8 million for men) [9]. In the largest five countries of the European Union (France, Germany, Italy, Spain, and the UK) plus Sweden (EU6) alone, the International Osteoporosis Foundation estimates that between 2017 and 2030, fragility fractures are expected to rise by approximately 23% [10]. Osteoporosis is a significant global public health concern in the elderly population. The cost of osteoporosis and related fractures is estimated to surpass $25 billion in the United States by 2025 [11]. The hip, forearm, clinical spine, and proximal humerus are the primary sites of osteoporotic fractures, accounting for around 80% of fracture-related injuries and contributing significantly to healthcare costs [12].

Bone is a composite of natural living tissue consisting of both an organic and an inorganic phase, the latter of which contains calcium crystals. Bone consists of approximately 60% inorganic minerals, 30% organic phase, and 10% water [13]. Bone strength is influenced by both the quantity and quality of the bone [14]. Bone quantity, commonly measured as bone mass or bone mineral density (BMD) [15], is widely used in clinical settings to estimate fracture risk. The arrangement and structure of trabeculae, which form the supportive framework of trabecular bone, play a crucial role in determining bone quality [16]. Moreover, bone tissue constantly remodels in response to mechanical loading, adapting its shape and redistributing weight to meet mechanical demands [17]. Factors such as the diameter distribution of collagen fibers, degree of crosslinking, fiber orientation, and age-related denaturation of collagen all significantly impact bone biomechanics. Additionally, the microscopic structure, mineralization level, and calcium-phosphorus ratio of hydroxyapatite (HA) crystals, and the distribution and degree of HA mineralization contribute to the overall performance of bone [18].

One of the primary risk factors for fragility fractures is reduced bone density. A decline in bone mass and lower bone quality contribute to increased bone fragility. In clinical practice, bone mass is commonly assessed to evaluate fracture risk, with BMD serving as a key diagnostic measure for bone fragility [19]. Dual-energy X-ray absorptiometry (DXA) is the preferred method for determining BMD [20]. It employs two X-ray beams of different energies, allowing the separation of bone from soft tissue and the calculation of bone mineral density. Similar dual-energy principles are also applied in CT and µ-CT [21,22]. However, DXA is limited in assessing bone quantity [23], and it primarily provides a two-dimensional (2D) projection of the bone’s three-dimensional (3D) structure [24]. Furthermore, the DXA technique does not provide information about trabecular bone microarchitecture, which is crucial for assessing fracture risk [25]. Consequently, DXA may incorrectly diagnose osteoporosis due to its inability to offer precise information regarding bone microstructure [26].

Bone exhibits a complex hierarchical organization, with mechanical behavior arising from features spanning multiple scales—from nanoscale collagen–mineral interactions to macroscale geometry [27]. This multiscale architecture governs bone’s mechanical integrity, which can be compromised by pathological conditions, particularly age-related diseases, at any level of the hierarchy [21]. Accurate fracture prediction, therefore, requires a comprehensive understanding of bone’s structure across scales [28]. Predictive tools are essential for both assessing bone fragility and developing bio-inspired design approaches in the production of bone scaffolding and implants [29,30].

Recent studies [31,32,33,34,35,36,37,38,39] have advanced our understanding of bone fragility, improved methods for estimating bone strength, and introduced novel techniques for its assessment. Despite these advances, predictive approaches remain fragmented, with limited integration across the molecular, cellular, tissue, organ, and whole-body levels necessary to capture the full complexity of bone mechanics. Multiscale analysis tools are essential for delivering precise and reliable assessments of bone strength and quality, which are critical for both prevention and treatment of bone disorders.

However, no comprehensive review has yet synthesized these predictive methods or explored how emerging multiscale tools could be integrated to enhance clinical decision-making. This review addresses that gap by:

1. Summarizing the current state of predictive tools developed across multiple biological scales.
2. Identifying gaps in their integration.
3. Outlining future directions for unified multiscale frameworks capable of more accurately evaluating bone fragility.

We focus on tools designed to assess fracture risk and mechanical performance from the molecular and cellular levels to the tissue, organ, and whole-body levels. The review also examines the clinical translation of these approaches for improved diagnosis of osteoporosis, fractures, and related pathologies, and highlights the main challenges to their integration.

Table 1 provides an overview of key predictive tools used at different scales. Moving forward, research must prioritize the development of cohesive multiscale frameworks that capture the complete spectrum of bone failure mechanisms—enabling earlier and more precise interventions for individuals at risk of fracture.

## 2. Recent Advances in Multi-Scale Predictors of Bone Fragility

**Figure 1:**
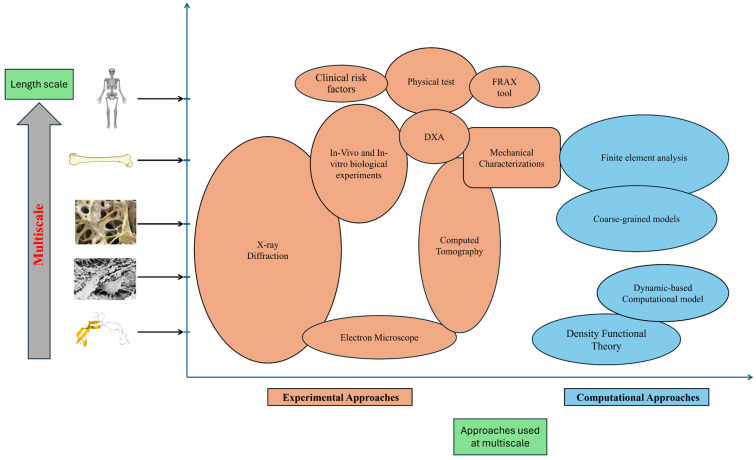
Multiscale predictive approaches (experimental and computational) that are used to analyze the bone fragility and strength at the different scales of the bone (molecular, cellular, tissue, organ, and whole body scale) (Derived from [21]).

Bone fragility is the intricate phenomenon that takes place across the various scales of the bone, ranging from the whole scale to its microscale constituents. In order to get complete information about the different scales of bone (molecular, cellular, tissue, organ, and whole-body level), which also significantly impact the fragility of fractures and strength, there is a need to analyze the bone at the multiscale level. The predictive approaches or tools that are used at the different scales of the bone to analyze bone strength or fragility are illustrated in Figure 1. Some of the distinct multiscale predictive approaches are elaborated below, highlighting their significance in assessing bone health and fracture risk. The summary of the different predictors that are used at the distinct scales for the analysis of the bone health assessment, primarily to estimate the risk of fractures or bone strength, is illustrated in Table 2.

### 2.1. Molecular Level

The diagnosis of osteoporosis, which leads to bone fragility, and its subtypes at the molecular scale must be required to find the exact problems and more efficient care plans for the patients [69]. Thus, early molecular intervention and diagnosis are of utmost clinical importance for individuals with osteoporosis diseases. Molecular predictive indices in bone health primarily focus on identifying and assessing specific biomarkers, genetic variants, and molecular pathways that influence bone strength, fragility, and the risk of bone-related conditions like osteoporosis. The genetic variables are essential in influencing bone strength and fracture fragility at the molecular scale. Many single-nucleotide polymorphisms (SNPs) linked to osteoporosis risk and BMD have been investigated by genome-wide association (GWAS) studies [70]. Numerous GWAS studies have investigated genes associated with osteoporosis, a condition commonly linked to aging. Ichikawa et al. [71] reported that premenopausal white and black women have a substantial BMD association with a single SNP. The results showed that the BMD of premenopausal white women was strongly correlated with two SNPs in the C6orf97/ESR1 area (*p* < 4.80 × 10^−4^), with suggestive evidence for CTNNBL1 and LRP5 (*p* < 0.01). Whereas, in premenopausal black women, there was additional evidence of relationship with one of the two SNPs in the C6orf97/ESR1 cluster. Kemp et al. [72] used quantitative heel ultrasonography to quantify BMD in a GWAS of 142,487 people from the United Kingdom Biobank. They discovered 307 conditionally independent SNPs at 203 loci that achieved genome-wide significance, accounting for around 12% of the variation in phenotype. After adjusting for multiple evaluations (*p* ≤ 1.6 × 10^−4^), they observed that 12 estimated BMD SNPs were linked to fracture. Their results demonstrate the substantial polygenicity of the genomic architecture underlying BMD. Furthermore, their investigations will be helpful in determining which proteins and pathways can be pharmacologically altered to reduce the prevalence of fractures in the general population.

In another analysis, Zhang et al. [70] observed that most of the reliable controlling SNPs related to osteoporosis from the 38 existing BMD GWAS studies used an unconventional epigenetics and transcription-based methodology. They discovered 14 new and reliable controlling SNPs (Tier 1 SNPs) for the possibility of osteoporosis among the >50,000 GWAS-generated SNPs. Their related genes, BicC family RNA binding protein 1, leucine-rich G protein coupled receptor 4, dishevelled-associated activator of morphogenesis 2, natriuretic peptide receptor 3, or high-mobility group AT hook 2, control cell signalling or enhancer activity and play a role in bone development or homeostasis. Furthermore, Liaw et al. [73] find the novel independently valuable SNPs linked to osteoporosis by using the Taiwan Biobank data. They used the data to determine and describe novel genetic variations connected to osteoporosis in Taiwanese people. The results showed that chromatin interaction modelling in mesenchymal stem cells linked this specific SNP to six genes, with a strong association with osteoporosis. The varying distribution of rs76140829 allele frequencies across different populations highlights the importance of considering population-specific genetic variations in osteoporosis research. These findings deepen the understanding of osteoporosis genetics, particularly in Asian populations.

Moreover, the receptor-activator of nuclear factor kappa beta (RANK), RANKL, and OPG are the signaling pathways that are involved in many other bone diseases, and their activity is either directly or indirectly regulated by the tumour cell to support its survival [74]. Bone metabolism regulation depends on the RANK/RANKL/OPG signaling axis. This system’s proper operation ensures an appropriate equilibrium between bone formation and resorption; when it is disrupted, many bone disorders that are characterized by fragility and an increased risk of fracture might result [75]. Ikebuchi et al. [76] discovered that the mature osteoclasts release vesicular RANK, which binds to osteoblastic RANKL and stimulates bone formation by activating Runt-related transcription factor 2 through RANKL reverse signaling. The RANKL–RANK system has increased significance for bone biology as a result of this discovery. Tourolle Duncan C. et al. [77], assessed the impact of therapy on μ-CT scans over time using in silico modeling based on pharmacokinetic and histomorphometry data. The findings were consistent with variations in bone volume fraction seen in biopsies taken during the tenth year of the FREEDOM Extension study from both the denosumab and placebo groups. They investigated that a little rise in the number of osteoclasts at the conclusion of the 6-month-dosing interval, particularly at the end of the extension trial, after long-term bone turnover suppression, resulted in increased RANKL production.

Another predictive approach used at the molecular level of bone health is the Wnt signaling pathway, which plays a crucial role in individuals with osteoporosis, as it is essential for bone growth and maintaining skeletal balance. In recent years, Lai et al. [78] created the novel assessment framework for osteoporosis in postmenopausal Caucasian women and pinpointed unique molecular clusters connected to the Wnt pathway. They compared the expression sequences of 12 genes linked to the Wnt pathway and osteoporosis among clusters 1 and 2 to find the variations in their activity. The finding investigated that PPP3CA, ITPR2, USP8, WLS, GSK3B, and CTNNB1 were highly expressed in cluster 1, while KLHL12 and PPP2R5B were up-regulated in cluster 2. Additional investigation revealed that most patients in cluster 2 were in the low BMD category, whereas most patients in cluster 1 were in the high BMD category. This innovative diagnostic method opens the door for early diagnosis, individualized treatment, and eventually better quality of life for osteoporosis patients, which may lead to a reduction in major fractures.

### 2.2. Cellular Level

At the cellular level, four primary cells, such as osteocytes, osteoblasts, osteoclasts, and extracellular lining cells, interact to create, control, and preserve bone [79]. The fragility of the bone at the cellular scale is analyzed using different predictive techniques to prevent the bone from a major fracture at the early stage through careful attention to the bone. Bone markers have been valuable in clinical practice, deepening our understanding of osteoporosis pathophysiology and the mechanisms of its treatment [80]. Bone resorption markers (elimination of ancient bone) and bone formation markers (establishing a fresh bone) are the two types of bone turnover indicators that are generated during bone remodelling procedures [81]. Osteoblasts generate or originate procollagen type 1 N-terminal propeptide (P1NP), osteocalcin, bone-specific alkaline phosphatase, and alkaline phosphatase as bone formation markers. On the other hand, bone resorption is indicated by the urinary N-telopeptides of type I collagen, tartrate-resistant acid phosphatase type 5 b, and serum C-telopeptides of type I collagen (CTX) [82]. The use of bone resorption markers is more common than that of formation markers. The marker levels can be used to determine both therapy responses and failures. Osteoporosis management can be improved by using biochemical indicators of bone turnover in conjunction with bone density tests [83]. Takada et al. [84] examined the assessment of the utility of P1NP on postmenopausal women (aged 55–90 years) having osteoporosis disease who previously consumed bisphosphonates were assigned to receive either teriparatide (20 µg once daily; *n* = 218) or open-label subcutaneous romosozumab (210 mg once monthly; *n* = 218). The result showed that P1NP is useful as a predictor of BMD reaction to teriparatide in practical patients who have never received treatment. In the teriparatide group of this examination, the predictive value of P1NP appeared to be impacted by prior bisphosphonate treatment. Another predictive tool at the cellular scale is carboxy-terminal Crosslinked Telopeptide of type 1 collagen (CTX-1), which is a reliable and highly sensitive biomarker of bone resorption that can be used to quickly assess how effectively postmenopausal osteoporosis is responding to bisphosphonate medication [85]. Whereas, Lee et al. [86] investigated the potential of using a specific blood marker of bone resorption to predict the risk of developing osteonecrosis of the jaw (ONJ) following oral bisphosphonate treatment. They evaluated the scientific validity of the morning fasting serum CTX test in 163 consecutive patients who underwent various oral surgeries, dividing them into two groups. Both groups were monitored for signs of bisphosphonate-associated ONJ for eight weeks post-surgery. The follow-up and medical records indicated that none of the patients developed ONJ during or after the observation period.

Furthermore, Altınsoy KE [87] examined the role of turnover markers such as n-telopeptide (NTX), C-terminal telopeptide (CTX), and deoxypyridinoline (DPD) in detecting osteoporosis and differentiating between affected bone and healthy bone. The study also evaluated the effectiveness of these biomarkers in monitoring treatment outcomes and predicting their success. The investigation was carried out between 1 January 2020, and 1 January 2023, for a three-year period. This extensive study led us to identify CTX, NTX, DPD, and tartrate-resistant acid phosphatase (TRAP) as critical biomarkers essential for assessing the condition of bones, tracking the efficacy of care, and identifying pathological fractures in the setting of osteoporosis. Consequently, CTX measures provided a valuable way to track patients with osteoporosis. Overall, this research highlights the dynamic changes in bone turnover markers following surgery, emphasizing the importance of monitoring and managing bone health during recovery. Furthermore, Bone turnover markers can improve prognosis by detecting disorders like osteoporosis at an early stage. Some bone markers showed better outcomes than others. The formation marker procollagen type 1 C-terminal propeptide (P1CP) is the most important in the osteoporosis scenario since it is a direct result of bone development. For the same reason, resorption marker CTX is also the most important, because it is also a direct result of bone digestion. These two markers (P1CP and CTX) distinguish from other, more complicated biomarkers due to their simpler analysis processes, which increase their dependability. Additionally, the increased bone turnover leads to decreased bone density and an increased risk of fracture or more severe injury [88].

### 2.3. Tissue Level

Recent advances in predictive techniques at the tissue scale have focused on enhancing the evaluation of bone density and microarchitecture to improve fracture risk estimation. Tissue-level tools assess the structural, morphological, and functional attributes of bone tissue that determine its strength, brittleness, and overall integrity. Considerable efforts have been devoted to incorporating these assessments into multiscale predictive models of bone fragility.

Among the most widely used tissue-level tools is the trabecular bone score (TBS), which estimates bone microstructure using two-dimensional DXA images. TBS is derived from the gray-level texture of lumbar spine DXA scans, where lower values reflect compromised bone structure characterized by reduced textural variation, greater amplitude, and a shallower slope. TBS has been shown to correlate strongly with key indicators of bone quality, including whole-bone stiffness, volumetric BMD (vBMD), cortical thickness, and trabecular parameters assessed by HR-pQCT. For example, Silva et al. [89] reported robust associations between TBS and multiple microstructural measures in patients with primary hyperparathyroidism, even after adjusting for body weight.

Age-related declines in TBS have been well documented. In a study of 129 participants aged 20–82.3 years, TBS showed a strong negative correlation with age (r = −0.55) and positive correlations with spine and hip BMD, as well as cortical and trabecular density and microstructure (*p* < 0.05) [90]. Large-scale registry data from Manitoba, Canada, further confirmed that TBS is an independent predictor of fracture risk, unaffected by prior or subsequent antiresorptive therapy [91]. The most recent version of the metric, TBS v4, incorporates DXA-based adjustments for soft tissue thickness, thereby improving accuracy across diverse populations [92]. Nevertheless, additional validation is required to establish whether it predicts major osteoporotic fractures as reliably as earlier versions, particularly in men, patients with type 2 diabetes, and those with hip fractures.

In addition to DXA-based indices, panoramic dental radiographs (orthopantomograms) have been extensively investigated as opportunistic tools for osteoporosis screening. Radiomorphometric indices, such as the mandibular cortical index (MCI), panoramic mandibular index (PMI), and mental index (MI), have shown good sensitivity and specificity in predicting low bone mineral density and osteoporosis-related fracture risk [52,53,54]. As panoramic radiography is one of the most frequently performed imaging modalities worldwide in dental and maxillofacial practice, it represents a cost-effective and accessible predictive tool. Its integration into multiscale diagnostic frameworks could enhance early detection of bone fragility, particularly in populations where DXA is not routinely available.

Beyond imaging, assessments of bone’s mechanical properties at the tissue scale offer complementary predictive value. Yeh and Keaveny [93] demonstrated that increased variability in trabecular thickness significantly reduces bone stiffness, independent of bone volume fraction, underscoring the contribution of microarchitectural heterogeneity to fracture risk. Such variability may be shaped by aging, disease, or therapeutic intervention. Nanoindentation techniques are frequently employed to quantify the local elastic modulus and hardness of bone, parameters that reflect microarchitectural integrity and material quality. In a study of osteogenesis imperfecta, Albert et al. [94] observed higher modulus and hardness values in type I patients compared with the more severe type III, highlighting the direct influence of disease severity on tissue-level mechanical properties.

Katsamenis et al. [95] examined cortical bone from young and elderly individuals, revealing that mechanical variability between lamellae and interlamellar zones at the osteonal level strongly correlates with fracture toughness and resistance to crack propagation. Reduced nanoelasticity heterogeneity was associated with lower tissue toughness and increased injury susceptibility.

Links between mechanical performance and BMD at fracture sites have also been demonstrated. In 2021, [96] examined human vertebral trabecular bone under near-physiological conditions and showed that QCT-derived BMD was a strong predictor of tissue-level material properties. At fracture sites, the mean BMD was 80.2 ± 28.7 mgCaHA/mL, confirming a clear association with mechanical behavior.

Further evidence comes from a µ-CT study of autologous bone grafts in 41 postmenopausal women undergoing spinal fusion and 26 men over 50 years old [97]. Postmenopausal women exhibited higher structural model index (SMI), reduced trabecular thickness (Tb.Th), and lower bone volume fraction (BV/TV) compared with men in the same age group. Among individuals with osteoporosis, men over 50 showed greater bone surface density (BS/BV), higher BV/TV, and thicker trabeculae than postmenopausal women. Overall, osteoporotic bone regardless of sex displayed substantially diminished quality compared with non-osteoporotic controls.

### 2.4. Organ Level

Advances in biomechanical modeling and imaging technologies have enabled more precise evaluations of whole-bone strength at the organ level. Predictive tools at this scale assess the bone’s structural integrity and mechanical performance under load, with applications such as QCT for volumetric density, DXA-based finite element analysis (FEA), and biomechanical strength indices (BSI). These approaches allow for the estimation of fracture risk or the early detection of fragility fractures before major breaks occur.

A two-dimensional FEA approach was validated by Den Buijs and Dragomir-Daescu [98] using cadaveric femurs. QCT-derived images were analyzed with FEA and compared to experimental measurements of femoral stiffness and fracture force. Digital image correlation combined with high-speed video recordings further enabled the computation of strain distributions.

Villamor et al. [99] proposed a semi-automated, patient-specific 2D FEA model based on DXA scans and medical records to assess hip fracture risk. Their study included 137 postmenopausal women (89 with fractures, 48 controls). The dataset was divided into a training set (*n* = 101) and a test set (*n* = 36). The model achieved an accuracy of 66.81% in training and 64.88% in testing, indicating moderate predictive power. Importantly, the method was shown to be fast, cost-effective, and adaptable to clinical workflows.

Hsieh et al. [100] developed a fully automated system that uses plain radiographs of the hip and spine for opportunistic assessment of osteoporosis and fracture risk. The predictive indexes achieved accuracies of 91.7% for hip osteoporosis and 86.2% for lumbar spine osteoporosis, demonstrating the potential of radiograph-based approaches.

More recently, the biomechanical strength index (BSI) has been introduced as a qualitative measure of bone fragility derived from FEA and DXA scans [31]. BSI quantifies mean strain distribution under simulated loading, with higher values indicating greater fracture susceptibility [48,49]. In a study of 846 women (mean age 60 years), BSI assessed at the spine, femoral neck, and hip was strongly associated with vertebral fracture risk. A cut-off value of 2.1 for spine BSI showed higher specificity and sensitivity for vertebral fractures compared with other fracture types [101].

Further validation came from an analysis of 31 L3 vertebrae donors (mean age 76 ± 10 years), where BSI correlated significantly with mechanical behavior (failure load, stiffness: r = −0.60, −0.59; *p* < 0.0001), DXA measures of aBMD and BMC (r = −0.93, −0.86; *p* < 0.0001), and microarchitectural indices such as trabecular bone volume fraction (Tb.BV/TV; r = −0.58, *p* = 0.001) and structural model index (SMI; r = 0.51, *p* = 0.004). These correlations remained significant even when controlling for aBMD [102].

Clinical studies have also highlighted the added predictive value of BSI. Ulivieri et al. [48] assessed 143 osteoporotic patients with spine X-rays and DXA-based BMD, TBS, and BSI at baseline and follow-up. Of the three indices, BSI was the most significant predictor of fracture risk, with higher values correlating with increased risk. BSI also showed a moderate positive correlation with SMI.

Finally, Ciao et al. [103] developed an FEA-based fracture risk prediction tool that incorporated DXA-derived aBMD alongside medical and demographic factors. This model was strongly associated with hip fracture risk in men, further confirming the clinical relevance of organ-level predictive approaches.

### 2.5. Whole-Body Level

The predictive techniques for the whole body concentrate on evaluating the overall condition of the skeleton, particularly bone mass dispersion, fracture risk, and musculoskeletal system integrity. Different researchers observed fracture risk and fragility fractures at the whole-body level using different predictive tools. Landi et al. [104] assess the level of physical activity in a European group of seniors receiving home care and investigate the connection between physical activity and disability incidents. More than half of the individuals engaged in physical activity, and 15% of patients experienced disability within the twelve-month follow-up period. Participants who reported engaging in physical exercises were significantly less likely to develop a disability than those with little to no physical activity. This finding remained consistent even after accounting for factors such as age, sex, and other potential confounding variables. To investigate the relationship between the physical activity elements and spatiotemporal gait metrics in older persons living in the community, Porto et al. [105] observed a connection between the components of structured physical exercise and spatiotemporal gait parameters by using a cross-sectional survey with 134 autonomous senior citizens. The assessments employed in this research were carried out on a single day by three qualified investigators: one for questionnaire administration and two for gait evaluation. The findings revealed that, among all the factors investigated in this study, active sport-based physical exercise may improve the overall gait efficiency of older people living in communities, regardless of prior practice. Here, the term ‘physical activity’ refers to general daily or lifestyle movements, while ‘physical exercise’ designates structured, intentional movement interventions.

In order to investigate the comprehensive assessment of bone fragility at the whole scale level, the fracture risk assessment tool (FRAX) was employed by incorporating multiple clinical risk factors [106]. FRAX, used to predict the risk of fragility fractures in patients with a 10-year follow-up period, was developed by the World Health Organization [107]. The following parameters are used to calculate the fragility fractures using FRAX: age, sex, body mass index (BMI), and dichotomized risk factors: rheumatoid arthritis, previous fragility fracture, parental history of hip fracture, smoking, ever use of long-term oral glucocorticoids, other causes of secondary osteoporosis, and excessive alcohol consumption [106]. FRAX estimates fracture risk for individuals between the ages of 40 and 90 using models that account for the risks connected with femoral neck BMD and clinical risk factors for fractures [108]. The FRAX algorithm produces two outputs: the 10-year chances of a MOF and the 10-year chances of a hip fracture [109]. MOF is a commonly and clinically significant outcome in fracture risk evaluation that is quantified with FRAX. Among the most frequent and dangerous fractures linked to osteoporosis, MOF includes fractures at the hip, clinical vertebrae (spine), distal forearm (wrist), and proximal humerus. Different countries use their own FRAXs at the whole-body level, incorporating country-specific epidemiological data to assess fracture risk more accurately. The Canadian FRAX was validated in the extensive cohort of 39,603 individuals, with a mean age of >65 years, and the majority of them were female obtained from the Manitoba Bone Density Program database that records all medical DXA examination outcomes in Manitoba, Canada. They investigated the FRAX probability based on the lumbar spine and femoral neck BMD T-score difference. The observed risk for osteoporotic fractures in men was 10.7% with a predicted value of 8.4%, whereas the 10-year estimate for all women was 12.0% with an anticipated rate of 11.1% for FRAX with BMD [110]. The study conducted by Sucharitpongpan et al. [111] examined the high risk of osteoporosis fractures in both men and women using FRAX. To forecast fragility fractures among the older adult population in Nan province, Thailand, the ideal FRAX-predicted 10-year probability of MOF thresholds values were 3.0% for men and 6.3% for women, as well as forecasting hip fractures, the ideal threshold values for FRAX hip fractures were 1.1% for men and 3.3% for women. These results indicate the optimal threshold FRAX values for men and women in hip fractures and MOF, which are used to identify elderly people of both genders who are at a higher risk of osteoporosis fractures. Cheung et al. [112] predicted the probability of fractures using the FRAX on the 2266 women’s samples. The average age of the women’s samples was 62.1  ±  8.5, and the average follow-up period was 4.5  ±  2.8 years. They investigated that as women grew older, their FRAX chances of developing osteoporosis and fragility fractures increased. Clark et al. [113] examined the high fracture risk in patients from Latin American countries by modifying the FRAX predictive tool. The results obtained using this predicted tool indicated that Ecuador had the smallest risk probability of a significant fracture, whereas Argentina had the greatest risk of the seven Latin countries. The investigations also showed that up to the age of 80, the risk chances for Brazil, Chile, and Mexico were comparable.

Moreover, Lesnyak et al. [114] created the FRAX evaluation and intervention levels for the Kyrgyz Republic, the Russian Federation, Uzbekistan, Armenia, Belarus, Georgia, Moldova, and Kazakhstan with the objective of improving medical care by utilizing the FRAX technique more widely to detect high-risk fractures in the bone. The investigations obtained using this tool revealed that for individuals aged forty, the probability was as follows: the lowest for Armenia, Georgia, and Belarus; intermediate values for Moldova and Uzbekistan; and the highest for Kazakhstan, Kyrgyzstan, and Russia. The clinical identification of individuals with a significant risk of fractures in males and females can be improved by implementing this novel FRAX-based intervention threshold, leading to higher healthcare frequencies. Meanwhile, for the ten Middle Eastern countries, Naseri et al. [115] developed the age-specific evaluations and intervention levels by utilizing FRAX. Notably, in Syria, there is the greatest variation in the risk probability of a significant osteoporotic fracture at 90 years old, with a difference of 18.2 among the lower and upper evaluation limits computed with FRAX (i.e., the upper threshold (43.20%) is 18.2% higher than the lower threshold (25%)). Conversely, the lower assessment threshold is defined as the age-specific 10-year fracture probability for a woman with a BMI of 25 kg/m^2^, without prior fractures or clinical risk indicators, and below which BMD testing and medical care are not deemed required. The upper evaluation limit, equivalent to the fracture probability for a comparable woman with a previous fracture and no BMD data, is determined to be 1.2 times the intervention threshold. On the other hand, Saudi Arabia has the tightest range at age 40, with a difference of only 0.52 between the lower and higher assessment standards.

Furthermore, Pluskiewicz et al. [116] analyzed the development of the FRAX, Garvan, and POL-RISK algorithms that can predict osteoporotic fractures and investigated the fractures in 457 samples of women using these developed tools. They discovered that only 11 samples with the observed fractures had a fracture probability greater than 10%, meaning that only 22.9% of women who experienced fractures (11 out of 48 patients) were correctly identified by FRAX as being at high risk. At present, Sheng et al. [117] compare the three different predicted tools, the osteoporosis self-assessment tool for Asians (OSTA), FRAX, and the one-minute osteoporosis risk test, on the 708 patients enrolled in Taiwan in 2010. Patients with injuries showed greater hip fracture risk scores, greater OSTA risk (5.9% ± 1.4% vs. 5.3% ± 1.3%), and higher FRAX MOF risk scores (14.0% ± 7.6% vs. 11.3% ± 5.7%) than patients without injuries obtained from the analysis. The result revealed that the only predictive tools that performed satisfactorily were OSTA and FRAX, highlighting the importance of paying attention when choosing prediction models for fracture risk evaluation. FRAX has been praised for its ease of use and suitability for primary care, but it has limitations, failing to account for exposure-response [118].

**Table 2:** Predictive tools and techniques for bone fracture assessment at the different scales.

Author	Tools/ Techniques	Samples Size	Population (Sex, Age)	Follow-Up Periods (Years)	Key Outcomes
Ulivieri et al. [48]	BSI	143	121 F and 22 M, ~60–67.9 y	-	The BSI hazard ratio of refracture with a 95% confidence interval was 1.201, and the *p*-value was 0.982−1.468.
Zhang et al. [70]	Transcription and Epigenetics	38 GWAS	-, -	-	The result indicated that from the 38 BMD GWAS, 14 osteoporosis-related control SNPs are linked to 5 genes.
Liaw et al. [73]	SNPs (GWAS)	29,084 used for GWAS and 18,918 for replications	-, -	-	One of the three SNPs found is rs78827626, which showed a lower certainty in its estimation with an information value of only 0.359.
Altınsoy K E, Unat B. [87]	CTX	520	-, ≥50	3	They found that CTX, NTX, DPD, and TRAP are important biomarkers that are essential for assessing bone health, tracking the efficacy of treatment, and identifying pathological fractures in the setting of osteoporosis.
Gama et al. [90]	HR-pQCT	129	M and F, 20–82.3 y	-	TBS and the trabecular and cortical bone characteristics determined by HR-pQCT showed a substantial correlation.
Xie et al. [97]	μ-CT	67	41 F and 26 M, >50	-	The result showed that Age and BMI weren’t significantly different between postmenopausal women and men over 50.
Villamor et al. [99]	DXA-based FEA	137	F, 81.4 ± 6.95 y	-	This novel approach brought around a 14 pp increase in precision compared to the gold-standard BMD.
Hsieh et al. [100]	DXA	5164 and 18,175 patients with pelvis/lumbar spine radiographs and Hologic DXA	77.4% of F in the hip evaluation and 79.6% in the spine evaluation set.	-	Comparing the developed tool to 3008 DXA performance during the identical investigation duration, 5206 (84.8%) subjects are classified as having a 95% either positive or negative outcome for osteoporosis.
Sornay-Rendu et al. [101]	BSI	846	F, 60 ± 15 y	-	There is 1.23 ± 0.28 Neck BSI, 1.13 ± 0.22 Tot Hip BSI, and 1.94 ± 0.60 Spine BSI observed using the developed predicted tool.
Landi et al. [104]	Physical Activity	2005	1559 F and 446 M, ≥65 y	1	Within the following twelve-month period, 15% or 370 participants experienced a disability.
Cheung et al. [112]	FRAX	2266	All F, 62.1 ± 8.5	4.5 ± 2.8 years	They observed the ideal cut-off value of 9.95% for FRAX with BMD.
Pulskiewicz et al. [116]	FRAX	457	F, 64.21 ± 5.94 y	10	The result shows that 6.3% for large FRAX fractures, 20.0% for any fractures in Garvan, and 18.0% for any fractures in POL-RISK.
Garnero et al. [119]	P1NP	473	M and F, 30–65 y	-	The observed bone formation marker has good efficiency and ease of use, which make it a potential tool for assessing osteoporosis victims.
Chalhoub et al. [120]	DXA	3301	All M, ≥65 y	-	The majority of fractures were linked to a higher risk when trabecular volumetric BMD and low aBMD were present.
McLean et al. [121]	DXA	1978	F, 50–93 y	8	The results indicated that women who have lesser lean mass as determined by DXA do not have a higher risk of hip fractures.
Messina et al. [122]	BSI	234	M and F, ~60–70 y	-	BSI hazard ratios with a 95% confidence interval were 1.372 and *p*-value 0.0261 for osteoporotic fragility re-fracture with the lumbar spine.
Qu et al. [123]	DXA	96 samples with OF and 107 samples with osteoporosis	All F, 74.8 ± 10.0 for OF and 71.5 ± 6.1 for non-fracture y	-	The findings demonstrated that the fracture sample’s BMD was smaller than the non-fracture sample.
Liu et al. [124]	μ-CT	105	44 M and 61 F, 43–71 y	Minimum 2	The result shows that the threshold value of BS/TV is 3.145, at which the surgeon can analyze the risk of fusion fracture in contrast to BMD evaluations.
Zellagui et al. [125]	Geometry + Mechanics Predictive Tool	636	-, 50–87 y	3 years	The result of the fracture risk obtained using this novel predictive tool is (True positive rate = 78%, True negative rate = 81%), which is much better than that obtained using DXA-based BMD.
Holloway et al. [126]	FRAX	591	All M, 40–90	-	There was no significant improvement in predicting fractures by MOF or hip FRAX with trabecular bone score modification.
Yang et al. [127]	DXA-based FEA	324 Prior hip fracture patients and 655 non-fracture patients	All F, ≥65	-	After changing for FRAX fractured hip possibility estimated using BMD, the increased hip danger of fracture was linked with lower FS and greater FRI.
Lee et al. [128]	μ-CT	30	-, -	-	The Tb.Th exhibited a slight difference irrespective of bone condition.
Liu et al. [129]	FRAX	1975	-, ≥40	6.8 ± 1.1 years	The measured to expected fractures proportion for MOF was 1.19 (95%CI 1.02–1.39) and for hip fractures it was 1.07 (95%CI 0.82–1.39) using the FRAX risk tool.
Li et al. [130]	QCT	1166	F, -	-	In older females with femoral neck fractures, osteoporosis may impact the association between proximal femur BMD and gluteus maximus muscle density.

## 3. Integration of Multi-Scale Predictive Tools for Early Diagnosis of Pathologies

**Figure 2:**
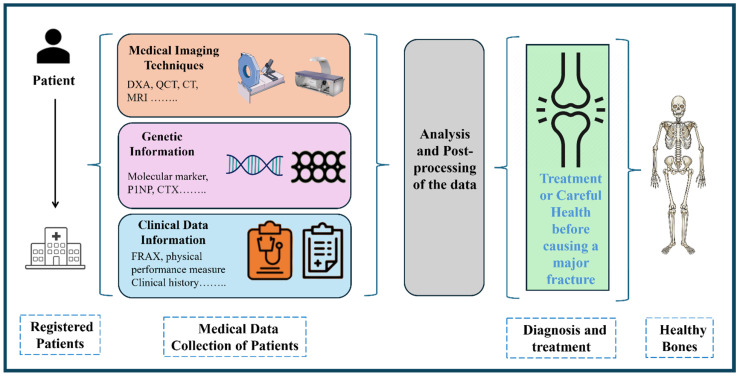
Procedure for integrating different predictive tools to analyze fractures at an early stage.

The integration of multiscale predictive tools for the assessment of bone fragility constitutes a critical methodological advance in the early diagnosis and management of osteoporosis and related skeletal pathologies. As discussed, bone fragility is a complex, emergent property arising from the interplay of structural, mechanical, and biological factors distributed across multiple hierarchical length scales—from nanoscale collagen-mineral interactions to organ-level load-bearing capacity. As such, effective predictive modeling of fracture risk necessitates a multiscale systems approach capable of capturing the interdependencies among these various determinants of bone strength (Figure 2).

At the molecular and nanostructural level, spectroscopic and imaging techniques such as Raman spectroscopy, nanoindentation, and synchrotron-based small-angle X-ray scattering (SAXS) provide critical data on mineral crystal size, orientation, collagen cross-linking, and hydration state [131,132,133]. These parameters directly influence tissue-level toughness and energy dissipation capacity. Cellular-scale information, including osteocyte lacuno-canalicular network (LCN) integrity, osteoblast/osteoclast activity, and signaling gradients, further modulates local mechanotransduction and bone remodeling kinetics, which are essential in maintaining mechanical homeostasis [79].

Integration begins with the acquisition of such heterogeneous data from distinct platforms and modalities. High-resolution imaging modalities—such as HR-pQCT, µ-CT, or ultra-high field MRI—enable the quantification of key morphometric parameters, including trabecular number, thickness, separation, and cortical porosity, which are strong determinants of local stiffness and failure load [64,89]. These structural features are then mapped to organ-level mechanical competence using subject-specific FE simulations, which estimate spatial distributions of stress and fracture risk under physiological loading conditions [134]. Crucially, the fidelity of these simulations is enhanced by incorporating tissue-level heterogeneity and anisotropy derived from imaging and histomorphometry.

At the systems level, clinical and biochemical data—such as serum markers of bone turnover (e.g., P1NP, CTX) [135], inflammatory cytokines, sex hormones, and comorbidities are integrated to contextualize localized fragility within an individual’s overall physiological and metabolic milieu. Machine learning algorithms, particularly deep neural networks [136], facilitate the integration and interpretation of these multiscale datasets by learning latent features and inter-scale dependencies that are predictive of fracture risk. These models are trained on annotated datasets and validated against prospective fracture outcomes to establish their diagnostic and prognostic performance.

A critical aspect of this integration is the harmonization of data across scales through multiscale feature extraction, dimensionality reduction, and standardized fragility scoring systems. For example, AI-enhanced low-dose chest computed tomography (LDCT) and DXA platforms can estimate volumetric BMD, and bone strength surrogates, while also incorporating image-based texture features indicative of trabecular disorganization or cortical thinning [137,138]. These predictions can be cross-validated and reinforced by outputs from complementary tools, such as IBEX Bone Health (IBEX BH) v1.3, which employs radiographic texture analysis to estimate regional areal BMD and infer microarchitectural degradation [139].

The convergence of these tools enables the construction of composite fragility indices that integrate microstructural, mechanical, and systemic predictors into a unified diagnostic framework. These indices are sensitive to early pathological alterations and thus capable of identifying high-risk individuals prior to the onset of clinical fractures. Moreover, such integrated models support in silico experimentation and digital twin frameworks, allowing simulations of disease progression and personalized responses to pharmacological or mechanical interventions.

Ultimately, the integration of multiscale predictive tools facilitates a transition from reactive to anticipatory bone health management, grounded in a quantitative, mechanistic understanding of fragility across biological scales. This approach not only enhances diagnostic accuracy and clinical decision-making but also paves the way for precision prevention strategies tailored to the individual’s unique biomechanical and physiological profile.

## 4. Translational Research and Clinical Applications

Building upon the integration of multiscale predictive tools for assessing bone fragility, a growing body of translational research is now focusing on adapting these advanced models and technologies for clinical application. While predictive tools operating at the molecular, cellular, tissue, organ, and whole-body scales have demonstrated substantial potential in research settings, their translation into practical diagnostic systems is essential to realize clinical impact. These tools offer the capacity to detect early microarchitectural or metabolic changes indicative of fracture risk—prior to clinical manifestations such as fragility fractures or osteoporosis—and thus facilitate timely, individualized interventions.

One notable example of such translational work is the proximal femur fracture index (PFFI) developed by Zellagui et al. [125]. This index integrates BMD, proximal femur geometry, and mechanical parameters derived from standard imaging data. It further incorporates anthropometric variables—such as weight, height, BMI, and center of gravity—as well as biomechanical fall-related variables, including estimated impact velocity during a fall from standing height. By combining these multiscale descriptors, the PFFI captures both intrinsic bone strength and extrinsic loading conditions, achieving a predictive accuracy of 78% for hip fractures. Importantly, this tool bypasses the need for complex finite element simulations, making it more accessible for clinical deployment. However, widespread clinical application requires the development of automated, robust segmentation algorithms for isolating the proximal femur from radiographic or CT data. These algorithms must ensure consistent extraction of cortical and trabecular regions, as well as anatomical landmarks, to support reproducibility across large populations.

In parallel, deep learning (DL) approaches are increasingly being developed to estimate BMD and fracture risk directly from routine imaging. Hung et al. [140] presented a DL-based two-stage framework wherein convolutional neural networks (CNNs) are trained to predict site-specific BMD values (lumbar spine, total hip, femoral neck) and corresponding T-scores using non-DXA images as input. In the second stage, predicted BMD values are combined with the FRAX score, excluding BMD to generate a final risk stratification. This hybrid model improves classification performance and supports early screening, especially in settings where DXA availability is limited. The authors demonstrated that DL-based BMD estimation could be used to prioritize patients for formal DXA scans, reducing the number of unnecessary referrals and optimizing resource allocation. The model is particularly effective in triaging individuals whose artificial neural network (ANN)-derived T-score falls below a predefined threshold or whose combined risk profile indicates elevated fragility risk. This two-tiered approach aligns with multiscale modeling by integrating organ-level bone quality (from predicted BMD), systemic clinical risk factors (from FRAX inputs), and machine-learned image-derived features.

**Figure 3:**
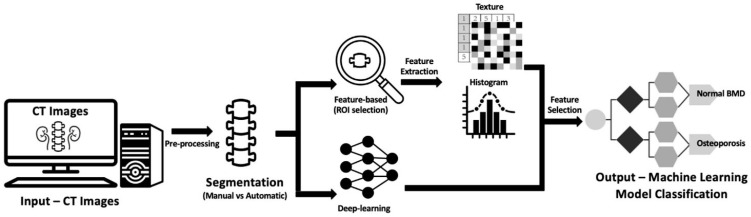
AI-based workflow for osteoporosis classification from CT images [141].

The growing relevance of DL for clinical application is further highlighted in the work of Ong et al. [141], who developed an AI-based osteoporosis classification system using CT imaging (Figure 3). Their pipeline consists of multiple stages, beginning with the acquisition of axial CT images, followed by preprocessing steps such as denoising, histogram equalization, and intensity normalization. Image regions containing trabecular bone are automatically segmented, either using thresholding techniques or with fully convolutional networks (e.g., U-Net architectures). Subsequently, high-dimensional radiomic features are extracted, encompassing texture descriptors (e.g., gray-level co-occurrence matrices), shape metrics, and histogram-based intensity features. These features are combined with clinical parameters (e.g., age, sex, BMI, comorbidities) in a supervised learning framework—typically a feedforward neural network or ensemble classifier such as gradient boosting—for final classification into normal, osteopenic, or osteoporotic categories. This integrative AI-based system enables personalized risk assessment based on bone texture and density heterogeneity, rather than relying solely on mean BMD values, and facilitates clinical decision-making by identifying patients who may benefit from early intervention or targeted therapies.

Further advances in clinical risk modeling are reflected in the recent launch of the FRAXplus Beta tool by the University of Sheffield [142]. This enhanced version of the standard FRAX calculator includes additional clinical risk modifiers such as TBS, type 2 diabetes status and duration, glucocorticoid dose, number of falls in the prior year, hip axis length, and the discordance between hip and spine BMD. These parameters reflect systemic and anatomical factors that modulate fracture risk beyond traditional BMD, enabling a more granular 10-year probability estimate for MOF. The FRAXplus Beta tool exemplifies the integration of systemic-level clinical data with organ-level geometric features, aligning with the multiscale framework discussed earlier.

Finally, radiograph-based predictive tools are emerging as accessible, low-cost alternatives to CT- or DXA-based methods. Hsieh et al. [100] developed an automated tool capable of detecting osteoporotic fracture risk from standard pelvic and spine radiographs. Their system applies image processing and ML techniques to predict BMD and fracture probability based on radiographic texture, bone shape, and cortical thickness. In a clinical deployment, the tool demonstrated high positive and negative predictive values and was able to opportunistically assess osteoporosis risk in 80% of individuals undergoing musculoskeletal radiography, many of whom had not received DXA evaluation. Unlike spine-focused CT tools, this method targets the hip and operates within existing hospital information systems, significantly reducing both radiation exposure and implementation barriers. This approach exemplifies scalable, population-level screening aligned with healthcare resource constraints.

Herein, these tools illustrate the accelerating translation of multiscale predictive frameworks into clinical practice. From rule-based indices and DL-assisted BMD prediction to AI-powered radiomic classifiers and enhanced clinical calculators, each application reflects an aspect of multiscale integration—whether at the biomechanical, morphological, or systemic level. The challenge moving forward is to establish standard operating procedures, harmonize outputs, and validate these models across diverse patient cohorts and imaging platforms. When deployed in concert, these tools promise to shift osteoporosis management from reactive fracture care toward proactive, personalized skeletal health strategies.

## 5. Conclusions

In conclusion, this study provides a comprehensive synthesis and critical analysis of both novel and widely adopted predictive tools used to assess bone fragility across multiple hierarchical scales. Multiscale approaches offer a more integrative and mechanistically grounded framework by capturing the interplay between microarchitectural changes, biomechanical properties, and genetic or systemic predispositions, spanning from the molecular and cellular domains to tissue, organ, and whole-body levels. Emerging and enhanced predictive tools—such as µ-CT, advanced derivatives of the FRAX algorithm (e.g., FRAXplus), and finite element analysis models based on DXA—demonstrate increased precision in evaluating fracture risk. By enabling early detection of structural weaknesses and high-risk patient phenotypes, these tools facilitate more effective preventive and therapeutic interventions. Nonetheless, many of these techniques have yet to be clinically validated on sufficiently large and diverse populations, limiting their translational impact. Therefore, future research must prioritize the optimization and standardization of these tools for routine clinical use, including large-scale validation studies and integration of high-resolution imaging and patient-specific data, to fully realize their potential in personalized bone health management.

## 6. Current Challenges and Future Directions

**Figure 4:**
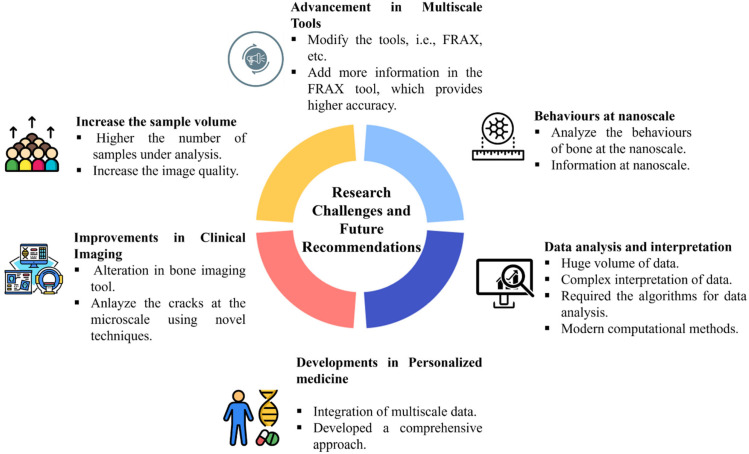
Research gaps and future recommendations in multiscale bone fragility analysis.

Despite substantial advancements in predictive tools for assessing bone fragility and fracture risk, significant barriers remain that limit their precision, clinical applicability, and translational impact. Addressing these challenges requires targeted efforts spanning fundamental research, computational modeling, imaging technologies, and clinical integration (Figure 4).

### 6.1. Key Barriers

A critical gap persists in understanding bone behavior at the nanoscale level. While experimental and simulation methods have elucidated bone structure and mechanics at micro- and macro-scales, the fundamental processes driving changes in bone mineral and organic matrix components with aging and disease remain inadequately characterized. Specifically, the non-collagenous proteins and nano-scale matrix organization that contribute to skeletal fragility and fracture risk during aging, are not fully understood [2]. These nanoscale changes critically influence the mechanical properties and toughness of bone but are often overlooked or insufficiently modeled in predictive frameworks. Consequently, predictive models lack the mechanistic depth required to capture early degenerative processes that precipitate fragility fractures.

At the clinical and tissue scale, widely used tools such as the FRAX algorithm exhibit substantial limitations. Although FRAX is designed for ease of use in primary care, its input parameters are primarily binary or categorical, neglecting dose or frequency-dependent effects of key risk factors such as glucocorticoid dosage, alcohol consumption, and number of prior fractures [143]. This simplification reduces the granularity and thus the accuracy of fracture risk estimates. Furthermore, FRAX does not fully incorporate emerging clinical variables such as bone microarchitecture or TBS, which have shown promise in improving fracture prediction [142]. The clinical utility of FRAX may therefore be restricted by insufficient parameterization and failure to capture patient-specific complexity.

Another significant obstacle is the limited size and heterogeneity of patient-specific imaging data available for computational model development and validation. Advanced FEA models and machine learning classifiers developed to predict fracture risk often rely on relatively small cohorts with variable image quality and acquisition protocols [99]. This constrains the statistical power and generalizability of such models and hinders their adoption in routine clinical workflows. Moreover, imaging datasets are often two-dimensional or low-resolution, failing to capture the three-dimensional microstructural features that govern bone strength.

In addition, current clinical imaging modalities, including DXA and conventional CT, lack the spatial resolution and contrast necessary to visualize fine microcracks and subtle changes in bone microarchitecture that precede fractures [141,144]. This deficiency limits early detection of fracture risk and prevents timely interventions. Although emerging modalities such as HR-pQCT and synchrotron radiation µ-CT offer enhanced imaging capabilities, their clinical translation is limited by cost, accessibility, and radiation exposure concerns.

Data integration and interpretation present further challenges. Bone fragility assessment increasingly involves multiscale data encompassing genetic, molecular, cellular, biomechanical, and clinical variables. The volume and complexity of these datasets are substantial, and extracting biologically meaningful and clinically actionable insights requires sophisticated computational frameworks [145]. Many existing machine learning algorithms lack interpretability, making it difficult for clinicians to trust and adopt them. Moreover, data heterogeneity and missing values complicate model training and limit robustness.

Finally, the full potential of personalized medicine in bone fragility management remains unrealized due to challenges in harmonizing multiscale data streams. Effective integration of genomic, proteomic, imaging, and clinical information is essential to develop individualized risk profiles and tailor preventive and therapeutic strategies [146]. However, current approaches are fragmented and rarely incorporate the diverse data types necessary for comprehensive precision medicine. This reduces the possibility for predictive models to capture the earliest degenerative changes that lead to fragility fractures, and more research is therefore needed in the field.

### 6.2. Potential Solutions and Future Directions

To overcome nanoscale knowledge gaps, future research should prioritize the development of integrated experimental and computational methods capable of characterizing and simulating bone behavior at the mineral and molecular levels. Advanced nanoscale imaging techniques such as atomic force microscopy (AFM) [147], nano-CT, and spectroscopy combined with molecular dynamics simulations can elucidate how hydroxyapatite crystal properties and matrix protein compositions evolve with aging and disease. Incorporating these findings into predictive models will enable mechanistic insights into the initiation of microdamage and fragility, bridging the current multiscale knowledge gap.

Additionally, improving clinical risk assessment tools demands the refinement of existing algorithms, such as FRAX, by incorporating dose-dependent risk factors and continuous variables. The ongoing development of FRAXplus, which integrates additional clinical parameters (e.g., fall history, diabetes duration, trabecular bone score), exemplifies efforts to enhance fracture risk stratification beyond binary inputs [142,143]. Machine learning methods trained on large, diverse clinical datasets can facilitate the modeling of complex nonlinear interactions among risk factors, providing individualized risk predictions with greater accuracy and clinical relevance.

Addressing data scarcity and heterogeneity necessitates concerted efforts to establish large-scale, standardized imaging databases encompassing diverse patient populations. Collaborative initiatives should promote the acquisition of high-quality, multimodal imaging data—such as 3D DXA, HR-pQCT, and MRI—alongside detailed clinical metadata. These datasets will enable robust training and validation of computational models, including patient-specific FEA and DL-classifiers that integrate biomechanical and clinical features [99].

Advancements in imaging technology and artificial intelligence are critical for overcoming limitations in spatial resolution and sensitivity. Emerging modalities such as HR-pQCT and synchrotron-based µ-CT can visualize bone microarchitecture and microdamage in unprecedented detail, but clinical translation requires optimization of protocols to reduce radiation dose and enhance accessibility [141]. Concurrently, AI-driven image processing pipelines can automate segmentation, feature extraction, and detection of microcracks, augmenting diagnostic accuracy and enabling early identification of fracture risk [144]. The AI-based osteoporosis classification workflow demonstrated by Ong et al. [141] illustrates the potential of combining CT imaging with machine learning for improved clinical decision support (see Figure 3).

To manage the complexity of multiscale data, developing interpretable machine learning frameworks and data integration platforms is paramount [145]. Methods such as explainable AI, multi-omics data fusion, and longitudinal modeling can improve clinicians’ trust and facilitate translation into practice. Computational tools capable of handling high-dimensional, multimodal datasets while preserving biological interpretability will enable precise identification of early microdamage and fracture precursors.

Finally, advancing personalized medicine in bone fragility requires comprehensive integrative platforms that unify genomic, proteomic, biomechanical, and clinical data. Such platforms, supported by AI models, can generate individualized fracture risk profiles and tailor interventions based on genetic predisposition, bone quality, lifestyle factors, and treatment response [146]. Collaborative efforts across computational biology, clinical research, and healthcare delivery are essential to develop, validate, and implement these precision medicine approaches, ultimately improving patient outcomes through targeted prevention and therapy.

Herein, the characteristics of an ideal predictive system for bone fragility can be envisioned as a multiscale, integrative framework that seamlessly connects biological, structural, and functional information across all hierarchical levels of bone. At the molecular scale, the system would incorporate genomic and proteomic data such as single-nucleotide polymorphisms, epigenetic markers, and biochemical turnover markers (e.g., CTX, P1NP), which provide early indicators of metabolic alterations preceding measurable bone loss. At the tissue level, advanced imaging modalities including DXA, HR-pQCT, and opportunistic panoramic radiography would be combined to capture quantitative features of bone density, trabecular microarchitecture, and cortical porosity. Organ-level data, such as geometry and strength indices derived from finite element analysis or biomechanical computed tomography, would then provide a direct estimate of load-bearing capacity and failure thresholds. Collectively, these data streams would be unified through AI-driven multimodal data fusion, enabling the construction of digital-twins—patient-specific computational models that continuously update as new clinical and biological data become available. Such digital-twins would allow real-time simulation of disease progression and virtual testing of pharmacological or lifestyle interventions, providing a highly personalized tool for predicting fracture risk and optimizing treatment strategies.

For such a system to transition from concept to clinical practice, several design principles must be prioritized. First, it must be clinically accessible, leveraging imaging modalities and biomarkers that are already widespread in routine healthcare (e.g., DXA, panoramic radiographs, serum bone turnover markers). Second, it must be scalable, capable of being deployed across different healthcare systems without prohibitive infrastructure demands. Third, it must be interpretable, providing outputs that clinicians and patients can readily understand and act upon, thus avoiding the ‘black box’ problem often associated with AI-driven solutions. Importantly, the predictive framework should be adaptive, incorporating feedback loops where treatment outcomes refine and improve future risk assessments. While cutting-edge technologies such as synchrotron-based µ-CT or ultra-high-resolution MRI will likely remain research tools due to financial, logistical, and geographic limitations, they play a critical role as benchmark platforms, validating the performance and accuracy of clinically feasible modalities. Ultimately, the realization of such an integrated system would mark a paradigm shift in bone health management, moving from reactive treatment of fragility fractures to anticipatory, precision-based prevention strategies tailored to individual patients.

## Figures and Tables

**Table 1:** Evaluations of the predictive techniques used to analyze bone fragility and strength.

Tools/Methods	Employed Scales	Predictive Indicators/Parameters	Effectiveness/Strength	Limitations	References
FEA	Cellular, tissue, organ, and whole scales, etc.	Maximum stress, Maximum strain, etc.	Simulates the complex loading conditions. Predict the entire model. Inexpensive relative to experiments.	FEA makes it more susceptible to misunderstandings, execution, and interpretation mistakes.	[40,41,42,43,44,45,46,47]
DXA + FEA	Organ and whole scale, etc.	Bone strain index (BSI)	Provide a better risk assessment of osteoporotic patients. BSI is suitable for irregular and complex structures.	Because of the characteristics of DXA images, the model reduces a 3D problem to 2D, which results in errors from thickness variability.	[48,49,50,51]
Panoramic radiography (Radiomorphometric indices)	Tissue/Organ	Mandibular cortical index (MCI), Mental index (MI), Panoramic mandibular index (PMI)	Widely available, low-cost, opportunistic use in dental practice	Limited standardization, variability in observer interpretation	[52,53,54]
CT-image based FEA	Tissue, organ, and whole scale, etc.	Stress (Maximum and minimum principal stress), strain (maximum and minimum principal strains), fractures, load, and sites, etc.	Vertebral fracture risk can be more precisely evaluated using new osteoporotic indices.	Routine clinical CT scans have limited resolution and higher noise, reducing the accuracy of bone geometry and material properties in finite element models.	[55,56,57,58,59,60,61,62]
µ-CT	Cellular and tissue scale, etc.	Trabecular Thickness (Tb.Th), Trabecular Number (Tb.N), Trabecular Separation, Trabecular Bone Volume Fraction (BV/TV), etc.	High-resolution morphological data are provided by CT. High resolution, relatively low cost, and scanning efficiency.	µ-CT has a low contrast sensitivity.	[63,64]
Peripheral quantitative CT (pQCT) and HR-pQCT	Tissue and organ scale, etc.	Volumetric Bone Mineral Density (vBMD), Cortical Thickness, etc.	Easy accessibility.	For the spatial resolution, only a few transversal data are available.	[65,66,67]
Biomechanical computed tomography (BCT)	Organ and whole scale, etc.	Hip BMD T-score, Femoral strength, Fragile bone strength, etc.	BCT analysis of routine abdominal or pelvic CT scans is as effective as DXA in identifying patients at high risk of hip fractures (BCT can identify patients at high risk of hip fractures).	BCT accuracy may be limited for scans acquired at lower kVp (e.g., 80 kVp) or reconstructed with sharp/nonstandard kernels.	[68]

## Data Availability

No new data were created or analyzed in this study. Data sharing is not applicable to this article.

## Abbreviations

The following abbreviations are used in this manuscript:

**Acronym**	**Meaning**
ANN	Artificial neural network
AI	Artificial intelligence
aBMD	Areal bone mineral density
AFM	Atomic force microscopy
BMD	Bone mineral density
BMC	Bone mineral content
BMI	Body mass index
BCT	Biomechanical computed tomography
BV/TV	Bone volume fraction
BSI	Bone strain index
CT	Computed tomography
COVID-19	Coronavirus disease 2019
CTX	Serum C-telopeptides of type I collagen
CTX-1	Carboxy-terminal crosslinked telopeptide of type 1 collagen
CTRA	Computed tomography rigidity analysis
CFS	Clinical Frailty Scale
CNNs	Convolutional neural networks
DXA	Dual-energy X-ray absorptiometry
DL	Deep learning
FE	Finite element
Fx	Fracture prediction
FRAX	Fracture risk assessment tool
FEA	Finite element analysis
GWAS	Genome-wide association studies
HRpQCT	High-resolution peripheral quantitative computed tomography
HA	Hydroxyapatite
IBEX BH	IBEX bone health
LDCT	Low-dose chest CT
LCN	Lacuno-canalicular network
MOF	Major osteoporotic fractures
µ-CT	Micro-computed tomography
OJN	Osteonecrosis of the jaws
OI	Osseous infarction
OPG	Osteoprotegerin
OSTA	Osteoporosis self-assessment tool for Asians
P1NP	Procollagen type I N-terminal propeptide
P1CP	Procollagen type 1 C-terminal propeptide
pQCT	Peripheral quantitative computed tomography
PHPT	Primary hyperparathyroidism
PFFI	Proximal femur fracture index
QCT	Quantitative computed tomography
RANKL	Receptor-activator of nuclear factor kappa beta ligand
RANK	Receptor-activator of nuclear factor kappa beta
SNPs	Single-nucleotide polymorphisms
SAXS	Small-angle X-ray scattering
SMI	Structural model index
Tb.N	Trabecular number
Tb.Th	Trabecular Thickness
TBS	Trabecular bone score
XFEM	Extended finite element method
YAM	Young adult mean
